# Optimizing Resources and Increasing the Coverage of Internet-of-Things (IoT) Networks: An Approach Based on LoRaWAN

**DOI:** 10.3390/s23031239

**Published:** 2023-01-21

**Authors:** Matheus Araujo Gava, Helder Roberto Oliveira Rocha, Menno Jan Faber, Marcelo Eduardo Vieira Segatto, Heinrich Wörtche, Jair Adriano Lima Silva

**Affiliations:** 1 Department of Electrical Engineering, Federal University of Espírito Santo, Av. Fernando Ferrari, 514, Vitoria 29075-910, ES, Brazil; 2 Sensors and Smart Systems Group, Institute of Engineering, Hanze University of Applied Sciences, 9747 AS Groningen, The Netherlands; 3 Department of Electrical Engineering, Eindhoven University of Technology, 5612 AZ Eindhoven, The Netherlands

**Keywords:** long-range wide area network, variable neighborhood search, minimum spanning tree, scalability

## Abstract

A resource optimization methodology is proposed for application in long range wide area networks (LoRaWANs). Using variable neighborhood search (VNS) and a minimum-cost spanning tree algorithm, it reduces the implementation and the maintenance costs of such low power networks. Performance evaluations were conducted in LoRaWANs with LoRa repeaters to increase coverage, in scenario where the number and the location of the repeaters are determined by the VNS metaheuristic. Parameters such as spread factor (SF), bandwidth and transmission power were adjusted to minimize the network’s total energy per useful bit ($E_{bit}$) and the total data collection time. The importance of the SF in the trade-off between ($E_{bit}$) and time on-air is evaluated, considering a device scaling factor. Simulation results, obtained after model adjustments with experimental data, show that, in networks with few associated devices, there is a preference for small values of SF aiming at reduction of $E_{bit}$. The usage of large SF’s becomes relevant when reach extensions are required. The results also demonstrate that, for networks with high number of nodes, the scaling of devices over time become relevant in the fitness function, forcing an equal distribution of time slots per SF to avoid discrepancies in the time data collection.

## 1. Introduction

The internet of things (IoT) presents itself as one of the main drivers in fifth-generation (5G) scenarios that demand low power and massive machine-type communications (mMTC) [1]. In the context of smart cities, IoT has driven the experimentation and the analysis of urban infrastructures, allowing innovation in mobility, energy systems, and healthy environments to serve the population in general [2]. Communication is a major concern in the connection of sensors, actuators, management platforms, and databases, to form IoT networks. Wireless communications are part of the solutions used to provide connectivity in such networks, bringing benefits that include the easy way of adding more devices [3]. Wireless sensor networks (WSNs) are successful in IoT deployments, despite the inherited challenges related to constraints of sensor nodes such as power consumption, computational capacity, bandwidth limitations, network management, and security [4]. Indeed, the trade-off that considers power consumption and relatively long reaches is a major concern of low-power wide area networks (LPWANs) applied in mMTC scenarios.

Among LPWANs, long-range wide area network (LoRaWAN) arose as an attractive solution for WSN deployments, in which end devices are power-limited and need to transmit a few bytes per transmission time [5]. Operating in the industrial, scientific, and medical (ISM) frequency bands, its physical layer standard, technically known as long range (LoRa), takes advantage of the modulation technique named chirp spread spectrum (CSS) to provide robustness against noise introduced in different types of environments and application classes [6,7]. The communication protocol implemented in LoRaWAN also helps to overcome IoT challenges related to long-range connectivity, as well as to reduced energy consumption, at the expense of low data rates. The authors of Ref. [7] show that LoRaWAN networks can establish connectivity of up to 15 km in line-of-sight links, according to data rates as low as 293 b/s.

LoRaWAN networks basically have three network components: end devices, Gateways, and a central server [8], End devices can only communicate with Gateways via single hop LoRa transmission and, on the other hand, Gateways can connect to the server via Ethernet, 4G, and 5G, among others [9]. Sensors and actuators are some of the typical battery-operated devices of the aforementioned networks. A LoRaWAN can play an important role in improving the battery life of such devices [10]. In fact, by adapting its main parameters and resources, the use of LoRaWAN can optimize the consumed energy [11]. One of the biggest challenges in the design of such WSNs relies on the development and deployment of intelligent solutions that are able to optimize resources, in particular, the ones related to energy savings [12,13,14]. In this context, we propose a methodology to optimize LoRaWAN parameters such as spreading factor (SF), bandwidth ($B_{w}$), and transmission power ($P_{t}$). A specific LoRa repeater device is required and considered in the proposed optimization algorithm to increase the range of the evaluated LoRaWANs.

### 1.1. Related Works

In Ref. [7], Bor and Roedig designed an automated mechanism for LoRa parameter selection in different transmission ranges. They reached link distances of 5 to 10 km with packet losses of around 40%. The authors of Ref. [15] presented an energy consumption model based on LoRa that allows power consumption estimation of each element of sensor nodes. Their model can be used to compare different LoRaWAN modes, aiming at the achievement of the best sensor node that enables network energy autonomy. Message replication and Gateways with multiple reception antennas were suggested in Ref. [16] to guarantee temporal and spatial diversities of LoRaWANs, respectively. Their results show that growths in user density and traffic are highly sensitive to message replication and, as expected, multiple antennas can improve network performance.

Numerical simulations were performed in Ref. [17] to gain an insight into the performance of LoRaWANs with different parameter settings. The authors show that even small adjustments in the medium access control (MAC) layer parameters can significantly affect the performance of the networks. A set of machine learning tools was used in the work described in Ref. [18] to configure LoRaWANs policies, and the results show an increase of up to 147% in the cumulative throughput per node. The authors of Ref. [19] presented a review of adaptive data rate algorithms for LoRaWAN with an optimization approach used to improve throughput, energy efficiency, and scalability; highlighting the strengths and drawbacks of each algorithm. Using a game-theory approach, the authors of Ref. [20] addressed the LoRa interference drawback that occurs when nodes are attached to a Gateway using the same SF in all uplink communications. Their results demonstrated that interference among end users impacts data rates in high-density networks. An adaptive solution was proposed in Ref. [21] to deal with the definition of the best LoRaWAN configuration, aiming at the minimization of channel utilization, and maximization of the number of delivered packages. The adaptive model was compared to a mixed integer linear programming model, and the results obtained from the adaptive heuristic were comparatively similar.

In Ref. [22], the authors suggested an optimization technique based on distributed genetic algorithm to improve performance via SF allocation. The goal was to maximize the packet reception probability, considering a restriction in the average energy consumed by all end devices. To provide reliable data transmission with low power consumption, the authors of Ref. [23] employed integer linear programming to configure nodes with optimal parameters. An adaptive dynamic network split mechanism was recommended in Ref. [24] for application in a LoRa-based smart city network, using maximum likelihood estimation to avoid resource scarcity. The recommended optimization model configures the SF and the $P_{t}$ parameters, focusing on the maximization of the performance utility in each group. The results show that the model adheres to the defined limits of quality of service in terms of delay, throughput, energy consumption, and reliability. The objective of the work presented in Ref. [25] was to find the ideal location of the Gateway in order to maximize the coverage probability. The addressed mixed-integer programming problem was decomposed into subproblems with a viable convex set. Considering solutions based on a gradient descent approach, the results show that the algorithm rapidly converges and the coverage probability is significantly improved when the Gateway location is optimized.

The impact of device scalability and densification on system reliability was evaluated in Ref. [26], considering LoRaWANs with multiple gateways. The proposed adaptive algorithm is able to optimize *SF*s by adjusting the signal-to-noise ratio thresholds, and therefore to improve throughput and packet delivery ratio. A different adaptive solution was evaluated in Ref. [27], defining the best LoRaWAN parameter settings to maximize the number of delivered packets. The results obtained by the developed heuristics were validated according to a comparison with the results provided by a mixed-integer linear programming solution. On the other hand, the evaluation described in Ref. [28] considered a multi-agent approach to allocate resources in multi-SF LoRaWANs, taking into account architecture design, logic implementation, and scalability. Regarding slot-length computation and end-node allocation strategies, the proposed approach resulted in network-size improvements of up to 66.7%. The authors of Ref. [29] developed an analytical model of LoRaWAN Class B mode that takes into account delay, data throughput, and energy consumption in downlink communication. With a specific cost function, they could establish a tradeoff between the waiting time of the frame in the gateway and the energy consumption of an end-device.

### 1.2. Paper Objectives and Contributions

It is worth mentioning that none of the above-mentioned works addressed the extension of coverage in an approach that considers a LoRa-LoRa repeater, as well as an optimization of the network resources using a variable neighborhood search (VNS) metaheuristic. To the best of our knowledge, this paper reports the first methodology useful in LoRaWAN coverage extensions provided by a LoRa-LoRa repeater, taking into account the optimization of some of the network resources, aiming at the minimization of implementation and maintenance costs. Therefore, it is important to mention that the performance evaluations were conducted in LoRaWANs, employing the aforementioned repeater to increase coverage with the minimum total transmission power and signal interference. The specification of the number of repeaters as well as their locations is part of the VNS metaheuristic roles. Hence, the contribution of this article is threefold: (*i*) we develop a simulation model that considers the connectivity between end devices, repeaters, and a Gateway, taking into account spread factor, bandwidth, and transmission power as optimization parameters; (ii) we perform simulation model adjustments with experimental results obtained in our previous work; and (iii) we optimized the LoRaWAN resources using graph theory and a VNS metaheuristic.

This paper is organized as follows. After the description of the coverage experiment and the required repeater in Section 2 and Section 3, we describe the adjusted system and the optimization models in Section 4 and Section 5, respectively. The performance analysis and the discussion are presented in Section 6 and Section 7, and the conclusion is provided in Section 8.

## 2. Preliminaries

**Figure 1:**
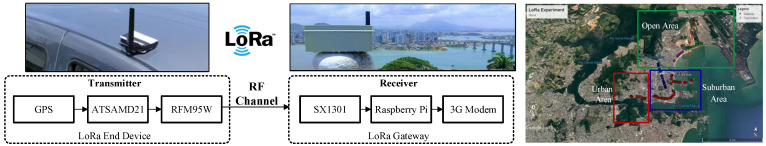
Experimental setup with photos of the used LoRa devices, and with a map of the areas selected for RSSI measurements. The operation frequency of the system was chosen between 902.3 to 903.7 MHz and the gain of the antennas was 3 dBi.

Figure 1 shows a block diagram of the setup we used in previous evaluations, aiming at simulation model adjustments. It also shows a map of the urban area (UA), the suburban area (SA), and the open area (OA) where the received signal strength indicator (RSSI) values were measured to substantiate the choice of the channel models used in the numerical model. The RFM95W LoRaWAN chip was used in the end device to send the transmitter coordinates obtained by the global positioning system module and processed by the ATSAMD21 microcontroller. The data stored in the database were collected by SX1301 LoRa concentrator, which is part of the Gateway that also comprises a Raspberry Pi micro-controller used to send the measured RSSIs via a 3G modem. The values of $P_{t}$, $B_{w}$ and SF used in the experiments were 20 dBm, 125 kHz, and both 7 and 12, respectively.

**Figure 2:**
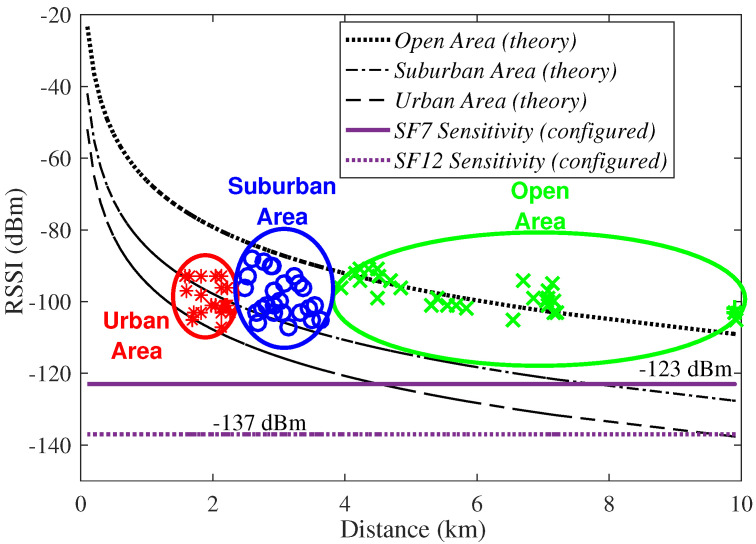
Values of RSSI measured at different distances and areas.

Figure 2 experimentally shows the RSSI values measured in the above-mentioned areas and, as consequence, several link distances. Figure 2 also shows comparisons between the practical measurements and the analytic Hata path loss approximations (in dB) given by

(1) $$\begin{array}{lll} PL_{UA}\left(d\right) & = & 69.55+26.16\times \log_{10}\left(f_{c}\right)-13.82\times \log_{10}\left(h_{TX}\right)-C_{RX}\\ & + & \left[44.9-6.55\times \log_{10}\left(h_{TX}\right)\right]\times \log_{10}\left(d\right); \end{array}$$

(2) $$PL_{SA}\left(d\right)=PL_{UA}\left(d\right)-2{\left[\log_{10}\left(f_{c}/28\right)\right]}^{2}-5.4;$$

(3) $$\begin{array}{ccc} PL_{OA}\left(d\right) & = & PL_{UA}\left(d\right)-4.78\times {\left[\log_{10}\left(f_{c}\right)\right]}^{2}-40.94+18.33\times \left[\log_{10}\left(f_{c}\right)\right]; \end{array}$$

for *d* the distance in meters, $f_{c}$ the center frequency in MHz, $h_{TX}$ the transmitter antenna height in meters and $h_{RX}$ the height of the receiver antenna. For small to medium-sized coverage, the receiver antenna correlation coefficient $C_{RX}$ is given by

(4) $$C_{RX}=0.8+\left[1.1\times \log_{10}\left(f_{c}\right)-0.7\right]h_{RX}-1.56\times \log_{10}\left(f_{c}\right),$$

and for larger coverage, it is given by

(5) $$C_{RX}=\left\{\begin{array}{c} 8.29\times {\left[\log_{10}\left(1.54\times h_{RX}\right)\right]}^{2}-1.1;\;if\;150\leq f_{c}\leq 200\;\mathrm{MHz}\\ 3.2\times {\left[\log_{10}\left(11.75\times h_{RX}\right)\right]}^{2}-4.97;\;if\;200\leq f_{c}\leq 1500\;\mathrm{MHz}. \end{array}\right.$$

The sensitivities for SF=7 and 12 provided by the device datasheet are, considering the Hata models above-described, represented in Figure 2 for sake of comparison. Figure 2 show that most of the RSSI measurements belong to Suburban Areas when a comparison with the Hata model is performed [9]. Nevertheless, all the above-mentioned areas can be distinguished from the measurements, taking into account some uncertainties in the classifications. The measured values are above the Hata path loss approximations considered in the Urban and Suburban Areas classification due to the mountain and the high buildings that exist in such areas, facilitating a line-of-sight transmission between the gateway and the itinerant end-device that increase the expected RSSI values. Considering the uncertainties, we can conclude that the channel models are suitable for the simulation model. Moreover, Figure 2 shows that SF must be increased for LoRa links greater than ≈4.5 km in UA and greater than 8 km in SA. The results also show that SF=12 should be employed in LoRaWANs of 10 km in UA.

## 3. The Demanded LoRaWAN Repeater

**Figure 3:**
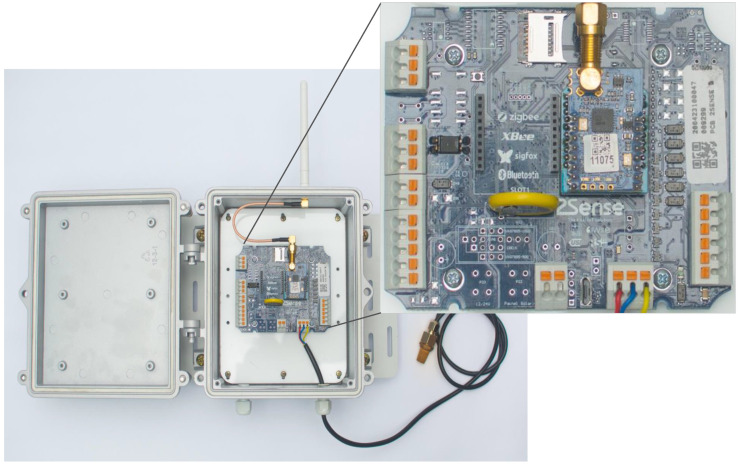
Multiple communication modules implemented in the Flex DAq device that can be used as a LoRaWAN repeater.

Figure 3 portrays a picture of the device named 2STools Flex DAq. Developed by Menno J. Faber under the supervision of Jair A. L. Silva and Helder R. O. Rocha, it is a multi-function device capable of operating as a data-logger, a basic control module, a wireless Gateway, as well as a repeater. It is part of a product family for automation systems and was designed for IoT applications and Industry 4.0 (http://www.2solve.com/site/en/technologies/iot-industry-4-0/, accessed on 1 December 2022). As a universal solution and independent of the market segment, this device was created to facilitate the automation of industrial processes. In customized demands, it is possible to carry out the integration of additional sensing modules that are directly controlled by a SamD21 ARM-M0 microcontroller through communication protocols such as SPI and UART.

The Flex DAq depicted in Figure 3 has multiple sensor inputs such as a pulse and frequency input, a Modbus RTU over RS485 or RS232, a load cell input, as well as three 24-bit analog inputs configurable between 4 and 20 mA or 0 and 10 V. It also has inputs for sensors such as PT100, PT1000 or thermocouple, and it is possible to connect digital sensors via I${}^{2}$C or One-Wire. The numerical model created in this work considers that this device is the applicable LoRaWAN repeater. Nevertheless, it is important to note that, beyond the LoRa connectivity utilizing the 915 MHz license-exempt band, it is possible to use other wireless communication standards such as Zigbee, Sigfox, Bluetooth and Wi-Fi, as shown in the zoom of Figure 3. For operation in different frequency bands, there is the ACW-LW8-EXT that, according to Ref. [30], allows LoRaWAN devices to be repeated in locations with poor network coverage such as underground parking or basement boiler rooms.

## 4. Simulation Model

**Figure 4:**
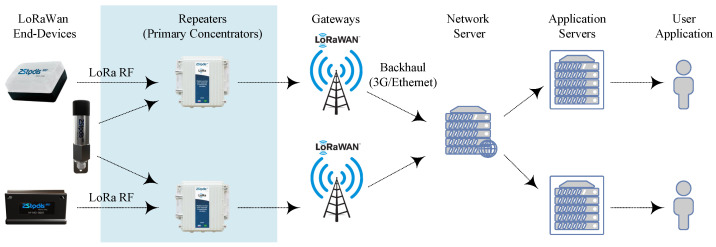
Structure of the proposed network for the optimization of LoRaWAN resources.

This section describes the simulation model based on the structure depicted in Figure 4. A metaheuristic was applied to optimize the resources of the extended LoRaWAN.

LoRa is the Semtech’s physical layer (PHY) solution that uses chirp spread spectrum as a modulation scheme. Its CSS model allows successful decodification with received signal powers below the noise floor, due to robustness against multipath fading losses, the doppler effect, and narrowband interference [31]. The range and the power consumption of LoRa-based LPWAN depend on the SF and $B_{w}$, as well as on the coding rate (CR) used in the forward error correction (FEC). Higher values of SF increase the signal time on-air (ToA), which means that more energy per transmitted bit is required for successful reception over longer distances. However, this reduces the effective system data rate. Hence, a trade-off between range and data rate is established, according to a predefined bandwidth. Channels with wide values of $B_{w}$ provide higher data rates but are more susceptible to noise, which limits the range. Additionally, LoRa employs FEC to increase reliability and range. However, the redundancy added in the FEC defined by the CR parameter slightly increases the ToA [31].

According to the authors of Ref. [7], a LoRa device can be configured considering one of the 6720 possible settings given by the combination of all possible values of SF, $B_{w}$, CR, and $P_{t}$. Thus, the choice of good sensor node parameters is essential in the design of a LoRaWAN, as this can result in their lifetime being 100 times shorter, making many commercial applications unfeasible [7]. Therefore, an algorithm capable of finding the ideal configuration of transmission parameters for each node is of paramount importance, in order to minimize capital expenditure (CAPEX) and operational expenditure (OPEX) of extended-reach LoRaWANs.

### 4.1. Configuration Parameters Used in the Model

Table 1 shows the parameters used to adjust the link performance and the power consumption in the proposed model [7]. The nominal system bit rate is obtained as

(6) $$R_{b}=SF\times \frac{B_{w}}{2^{SF}}\times CR.$$

### 4.2. Packet Structure

A LoRa packet starts with a preamble used for time synchronization, followed by an optional header that carries the payload size and LoRa configuration data. The header is always encoded with a CR=4/8, whereas the payload is encoded with a variable CR. A cyclic redundancy check (CRC) can be added at the end of the frame [11]. For an observed payload (PL) in bytes, the number of symbols ($N_{\mathrm{payload}}$) used to transmit the useful payload can be obtained as

(7) $$N_{\mathrm{payload}}=8+\max\left(ceil\left(\frac{\Theta (PL,SF)}{\Gamma (SF)}\right)\times \frac{1}{CR},0\right),$$

in which

(8) Θ(PL,SF)=8×PL−4×SF+16+28−20×H

and

(9) Γ(SF)=SF−2×DE.

In Equation (9), DE is equal to 1 when data rate optimization is enabled and 0 otherwise. The parameter *H* of Equation (8) is equal to 0 when the header is enabled and 1 otherwise. The ToA is given by the sum of the preamble duration ($T_{\mathrm{preamble}}$) and the payload duration ($T_{\mathrm{payload}}$) as follows

(10) $$\mathrm{ToA}=T_{\mathrm{preamble}}+T_{\mathrm{payload}},$$

in which $T_{\mathrm{preamble}}=\left(4.25+N_{p}\right)\times T_{\mathrm{symbol}}$ and $T_{\mathrm{payload}}=N_{\mathrm{payload}}\times T_{\mathrm{symbol}}$, considering $T_{\mathrm{symbol}}=2^{SF}/B_{w}$ the CSS signal duration.

### 4.3. Energy Consumption Model

The authors of Ref. [11] proposed the following energy consumption per useful bit ($E_{bit}$) metric used in this work to evaluate the consumption of the LoRaWAN end devices

(11) $$E_{bit}=\frac{P_{cons}\left(P_{t}\right)\times \left(N_{\mathrm{Payload}}+N_{p}+4.25\right)\times 2^{SF}}{8\times PL\times B_{w}},$$

where $P_{cons}\left(P_{t}\right)$ is the power consumed by the end device that depends on the transmission power.

## 5. Optimization System Model

The optimization proposed in this work considers that the end devices are connected by wireless links using LoRa, and the repeaters are able to receive LoRa signals from end devices and repeat them to a Gateway. In addition, there is an energy management system where the main Gateway should be located, and an area of 10 km × 10 km = 100 km^2^ was delimited for the device allocations. The area was divided into different propagation environments, named urban, suburban, and open areas, before the definition of the number of allocation points. Then, we empirically defined that 70% of the points should be located in the urban area, 20% in the suburban area, and 10% in the open area. It is worth mentioning that, initially, the points are randomly allocated in each area, as well as the height of the antenna installed in each point, which randomly assumes integer values between 2 and 40 m. The payload size defined for each installation point is also randomly assigned and can vary from 10 to 80 bytes in steps of 5 bytes.

The methodology seeks to find which device should be installed at each point and optimize resources using the configuration parameters $B_{w}$, SF, and $P_{t}$, in order to establish the best links. It also provides a scaling factor for each device, i.e., it defines when each end device or repeater is able to perform its uplink with minimum interference and, consequently, with less power consumption.

The network was modeled by a graph G=(V,E), in which *V* is the set of sensor nodes, a Flex DAq repeater and the Gateway, and *E* is the set of edges that indicate the links. A structure for the network with two-level links is proposed, in which the first is composed of an end device and a repeater, and the second of a repeater and the Gateway. Each link considers an associated non-negative cost c(u,v) designed by the propagation losses defined by the Okumur–Hata method, and then the adjacency matrix of the graph *G* is calculated. The matrix data receive null values when there are no links and non-null values when there is a link between any two vertices of the graph. The adjacency matrix of the modeled network is a square matrix, meaning that the number of lines is equal to the number of columns, which is equal to the number of devices in the network.

### 5.1. Definition of the Initial Solution

The definition of the initial solution problem is done through the Prim algorithm [32]. It receives the adjacency matrix of graph *G* as input and outputs the minimum spanning tree (MST) of *G*. The MST is one of the most important challenges in graph theory. Several applications use this concept in the design of transport and telecommunication networks, where the problem is defined in a graph G=(V,E), for *V* the set of vertices and *E* the set of undirected edges with associated non-negative costs. An MST is defined as a connected subgraph of *G* without redundancies, passing through all vertices in *V* with the minimum cost [32]. In this work, we implemented the Prim algorithm to solve the MST problem for an undirected connected and weighted graph. It is inherently greedy and random as it starts at a random node of the graph and, in each iteration, examines all available edges from visited to unvisited nodes to choose the one with the lowest cost. Then, the chosen edge is added to the set of visited nodes and the graph edge is added to the MST. In Algorithm 1, this procedure is systematically described, for $T_{min}$ the set of edges that define the MST, *T* and *M* are the sets of selected and not selected vertices, respectively.
**Algorithm 1** Pseudo-code of Randomized Prim**Require:**G=(V,E) and adjacency matrix $D=d_{ij}$
  for all edges i.j;
1:Choose any vertex i∈V2:$T\overset{}{\leftarrow }\left\{i\right\}$;3:$M\overset{}{\leftarrow }V\backslash \left\{i\right\}$;4:$T_{min}\overset{}{\leftarrow }\emptyset$;5:**while**∣T∣≠∣V∣**do**6:   Find an edge (j,k)∈E such that j∈T,k∈M   and $d_{jk}$ is minimum;7:   $T\overset{}{\leftarrow }T\cup \left\{k\right\}$;8:   $M\overset{}{\leftarrow }M\backslash \left\{k\right\}$;9:   $T_{min}\overset{}{\leftarrow }T_{min}\cup$ (j,k);10:**end while**11:**return**$T_{min}$;


We modified the Prim algorithm to limit the depth of the MST to two due to the considered two-level link structure. The modification was done as follows: after selecting the minimum-cost edge to be added to the set of edges, a depth check of the resulting tree is made and if the depth is greater than two, this edge is discarded and a new edge is chosen. This tree became the starting point of the optimization algorithm. Finally, with the definition of the links by the Prim algorithm, values of SF, $B_{w}$, and $P_{t}$ are attributed to each node to define the initial solution. Initially, the values of SF and $B_{w}$ were, for all nodes, fixed in 12 and 125 kHz, respectively. The values of $P_{t}$ are evaluated in this procedure considering the following link budget equation

(12) $$P_{r}=P_{t}+G_{sys}-L_{path},$$

in which $P_{r}$ is received power in dBm, $G_{sys}$ is the total gain of the system in dBi, and $L_{path}$ is the link attenuation in dB obtained with the Okumura–Hata model for urban ($PL_{UA}$), suburban ($PL_{SA}$), and open areas ($PL_{OA}$). For the determination of $G_{sys}=6$ dBi, it is considered that the transmitter and the receiver antennas have gains equal to 3 dBi. We also deliberated that $P_{r}$ should be 98% of the sensitivity (*S*) of the end devices. The sensitivity is defined as the lowest signal strength that allows message decodification at the receiver. Here, *S* is computed as

(13) $$S=-174+10\times \log_{10}B_{w}+NF+SNR,$$

for SNR the signal-to-noise ratio. It should be noticed that, in this work, the sensitivity is closely related to RSSI.

### 5.2. Optimization of Resources

Variable neighborhood search (VNS) is a metaheuristic that changes neighborhood structures to find local minimal solutions, as well as to escape from the current local minimal aiming at a better solution. Usually, the steps used in the basic VNS (BVNS) are a disturbance of the current solution, local search, and change of neighborhood. Generally, the type of search used in a BVN is known as the first enhancer. The pseudocode presented in Algorithm 2 illustrates the steps of a BVNS.

In this work, the BVNS was adapted as follows: a random solution is generated for each visited neighborhood structure, which may refer to a non-connection network. A network is considered non-connected when at least one of its devices, even configured with its highest transmission power, is not able to offer connectivity between its pairs, according to the current values of $B_{w}$ and SF. Thus, the local search explores the set of solutions defined by that neighborhood, to bring back the modeled connectivity. The BVNS remains in the same neighborhood structure until a network-connected solution is defined. When a better solution is founded, the algorithm leaves the explored neighborhood structure and immediately returns to the first structure that is designated to explore or remains in the current structure with the new incumbent solution. Otherwise, it will proceed to the next neighborhood structure until all structures in the neighborhood are visited.
**Algorithm 2** Pseudo-code of a BVNS**Require:***s* (Initial Solution), $k_{max}$ (number of neighborhood structures), Stop_Condition.
1:**while**Stop_Condition is not met **do**2:   k←1;3:   **repeat**4:     $s^{\prime }\leftarrow$Shake(s,k);5:     $s^{\prime \prime }\leftarrow$Local_Search($s^{\prime }$);6:     s,k←Neighborhood_Change(*s*, $s^{\prime \prime }$, *k*);7:   **until** $k=k_{max}$8:**end while**9:**return***s*;


Neighborhood structures are used to promote perturbations in the current solution aiming at the generation of another. Here, the neighborhood of a solution is characterized by systematic modifications in the structure of the communication links and in the configuration parameters of the devices. It is important to emphasize that the disturbances experienced by the current solution do not result in changes in the physical topology of the graph that represents a network under evaluation. Hence, in this context, four neighborhood structures were defined to be used in conjunction with the proposed optimization algorithm. They are carried out from first to last, according to a proper requisition. The neighborhood structures defined in this work are:

- Randomly chooses a network point and assigns a pair of SF and $B_{w}$ to it;
- Exchanges the root of the tree with another randomly chosen network point;
- Changes a connection in the graph, randomly chooses a level 3 point (end-device) and connects it to another point of level 2 (repeater) or level 1 (Gateway);
- Chooses any point of level 2 or 1, that has an associated schedule of transmission; select the SF of this schedule that has the largest number of allocated time slots; randomly chose a node with this SF and assign it a different SF value.

### 5.3. The Objective Function Considered in this Work

The minimized objective function (OF) takes into account the ($E_{bit}$) metric (see Equation (11)) and the total network data collection time that is related to the ToA parameter. Delivering a large number of packages in a small amount of time becomes a costly task due to the increase in possible collisions between packages. To solve this problem, we proposed an optimization algorithm in which a schedule of transmissions from end devices to devices of levels 1 and 2 are considered. Broadcasts with the same SF are scheduled into different slots to avoid collisions, while those with different SFs can occur in parallel. Therefore, each repeater and Gateway will have a transmission schedule of the nodes that are connected to them. Each link will have its respective ToA, which means that the optimization should minimize the maximum ToA of each schedule through a systematic allocation of the SF, thus reducing the total data collection time of the network. The modeled OF is represented by Equation (14) in which *N* is the number of network devices and *L* is the amount of scheduled node transmission, i.e., the number of repeaters plus the Gateway.

(14) $$OF=\sum_{i=1}^{N}E_{bit,i}+\sum_{j=1}^{L}ToA_{max,j}.$$

## 6. Performance Evaluation of the Optimization

**Figure 5:**
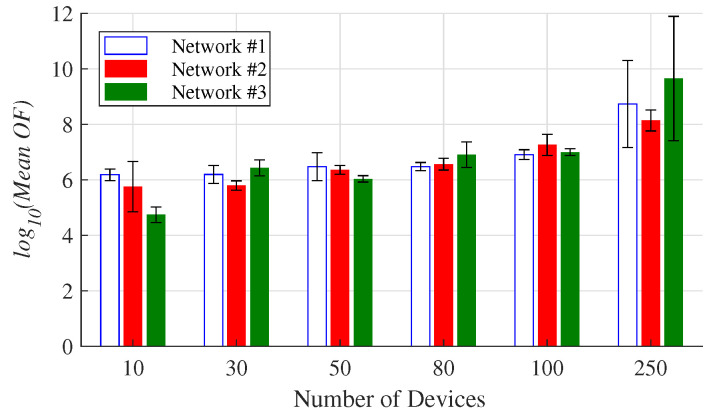
Average OF obtained in six of the different analyzed scenarios.

The evaluations were performed using Matlab and an Intel(R) Core(TM) i5-9300H computer with a 2.4 GHz CPU and a RAM of 8 GB RAM. To test the proposed method, 3 networks named Network #1, Network #2, and Network #3 were evaluated 6 times, in 10 scenarios with 10 (S10), 30 (S30), 50 (S50), 80 (S80), 100 (S100), 130 (S130), 160 (S160), 190 (S190), 230 (S230), and 250 (S250) devices. This means that in the scenario S10, Network #1, Network #2, and Network #3 are able to randomly install equipment in 10 locations, whereas in S30 they can do it in 30 locations, and so on. As mentioned above, the random allocations should take into account the following rules: 10% of the locations belong to open areas, 20% to suburban areas, and 70% to urban areas. Moreover, the height of the antennas is also randomly chosen between 2 and 40 m. Figure 5 shows that, on average, the logarithm of the OF linearly grew with the size of the networks.

**Figure 6:**
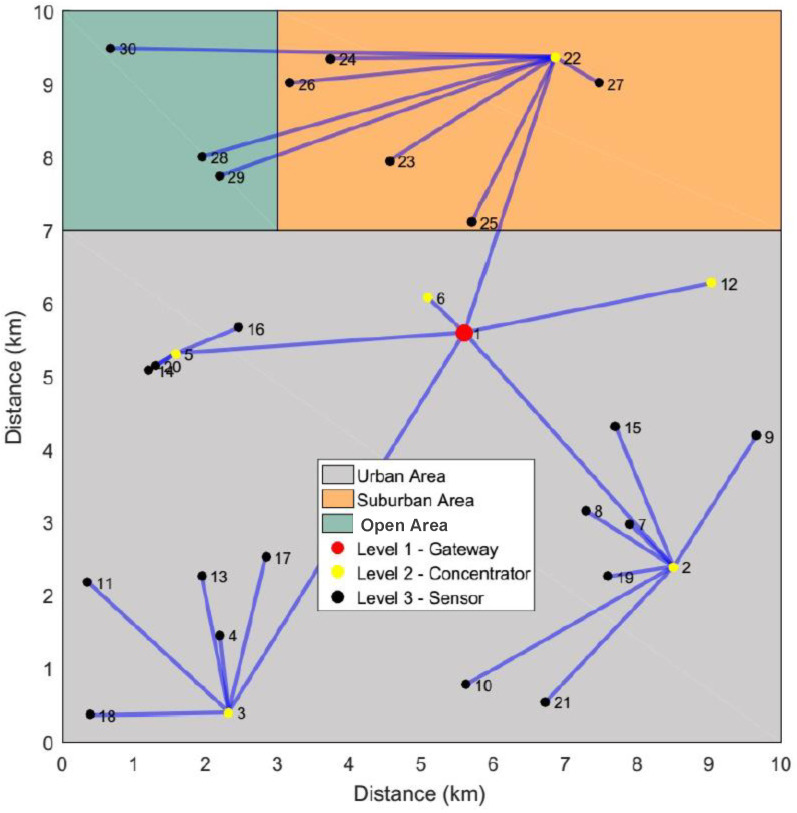
Optimal solution for the points allocated in the Network #1 with 30 devices. The concentrator is a data repeater and the sensor is an end-device.

The standard deviations obtained in the Networks #1 and #3 at the S250 scenario (see Figure 5) indicate that, for the same networks, the algorithm can provide different topologies in terms of connectivity. This is due to the stochastic nature of the algorithm, which allows the reach of different local optimums during resource allocation. This reinforces the idea that the algorithm must be executed more than once in the same instance. The optimal allocation for the Network #1 with 30 devices is geographically represented in Figure 6.

Figure 6 shows that a single repeater (concentrator) is able to connect all end-devices allocated in the Suburban and Open Areas, whereas three are required to attend to the connectivity demanded by the Urban Area. It can also be observed that, in this evaluated scenario, the sensor allocations demanded in the Open Area uses the Suburban area repeater to connect with the Gateway. It is worth commenting from Figure 6 that at Level 2 an end-device (terminal node) should be allocated if no repeater allocation is recommended by the optimization procedure at this level.

### 6.1. Evaluation of $E_{bit}$, $P_{t}$ and Transmission Rate ($R_{b}$)

**Figure 7:**
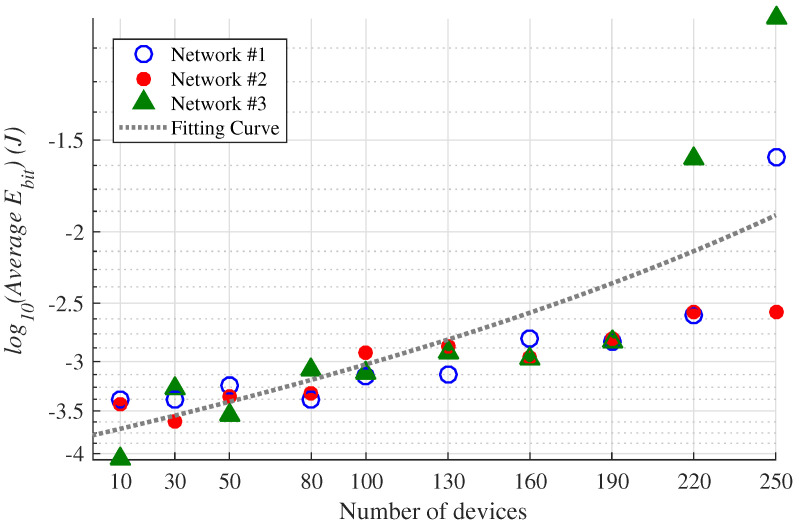
Total average $E_{bit}$ obtained in all evaluated scenarios. The fitting curve was obtained using the quadratic polynomial curve *Poly2* described in Ref. [33].

The energy consumed by devices ($E_{bit}$) is one of the main available resources that must be well-planned to maximize the lifetime of the network elements. Figure 7 shows the obtained total average $E_{bit}$ of the networks, in each scenario. The fitting curve was obtained using the quadratic polynomial curve *Poly2* described in Ref. [33].

Figure 7 shows that, as expected, the greater the number of devices in the network, the greater is the total average $E_{bit}$, since it is the sum of the average $E_{bit}$ of each device. However, added to this fact, we have the time scaling factor of the devices considered in the OF. Thus, as the number of devices increases, the algorithm tends to evenly distribute the devices among the available SF values, no longer giving preference to smaller SFs in order to reduce the total average $E_{bit}$. Therefore, as the number of devices in the network increases, the use of larger values of SF is more common, causing considerable increases in the $E_{bit}$ related metric. Furthermore, it can be verified in Figure 7 that networks with the same number of devices, which require different parameter configurations, provide a different order of magnitudes in total average $E_{bit}$. The differences in the networks of the same number of devices arise due to different design needs, which are related to geographical allocations, transmitter heights, receiver antennas, as well as requirements for the size of the data to be sent to each point. These design requirements are translated by the optimizing algorithm into the parameters $P_{t}$, $B_{w}$, SF, and PL, which influence the $E_{bit}$ related metric.

**Figure 8:**
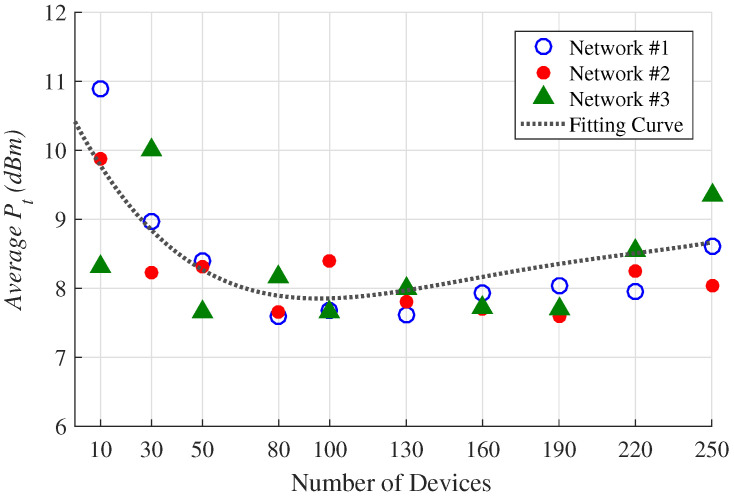
Average $P_{t}$ obtained in all evaluated scenarios. The fitting curve was obtained using the quadratic polynomial curve *Poly2* described in Ref. [33].

Figure 8 shows the normalized average transmission powers provided to each considered network and scenario. It can be observed from Figure 8 that the smallest allocation of average $P_{t}$ occurred in the networks with a number of devices between 80 and 100, with powers between 7.81 and 7.91 dBm. Therefore, in terms of this paramount parameter, the networks that have this amount of components have the best cost-benefit. Figure 8 also shows that the required values of $P_{t}$ increase with the decrease of the network devices. This is explained by the fact that, in networks with few elements, the link distances are relatively large. This increases the propagation losses and, consequently, the transmission power required by the devices. Apparently, this issue can be overcome by increasing the SF. However, in this case, the penalization provided by the OF is greater, when compared to the increase in $P_{t}$.

**Figure 9:**
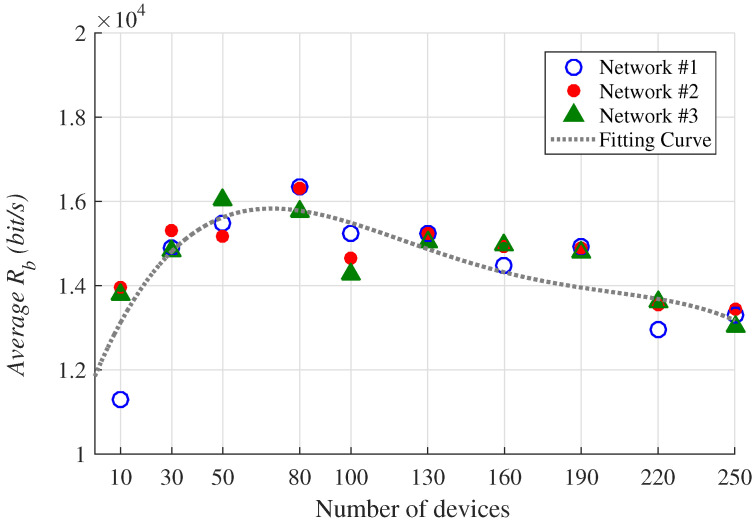
Average values of $R_{b}$ obtained in the analyzed scenarios. The fitting curve was obtained using the quadratic polynomial curve *Poly2* described in Ref. [33].

Figure 9 shows the average values of $R_{b}$ obtained in the analyzed scenarios. It should be stressed that in these evaluations, CR was fixed at a value equal to 4/5. Thus, considering Equation (6), the maximum transmission rate that can be achieved by the optimization procedure is $R_{b}=21.875$ kb/s, when $B_{w}$ and SF are 500 KHz and 7, respectively.

It can be seen in Figure 9 that the highest average $R_{b}$ provided by the optimizer was obtained in networks with 80 devices, being equal to 16.15 kb/s, which is ≈73% of the maximum rate. The lower values achieved in networks with 10 devices are due to greater link distances that demand larger values of SF to maintain the connectivity, to the detriment of $R_{b}$. Nevertheless, in the networks with 250 devices, the distance of the links is no longer a predominant problem, but rather the scaling of devices with practically all available SFs being used for data transmission. Indeed, this strategy minimizes the OF at the cost of reductions in $R_{b}$.

### 6.2. Evaluation of Device Scaling

**Figure 10:**
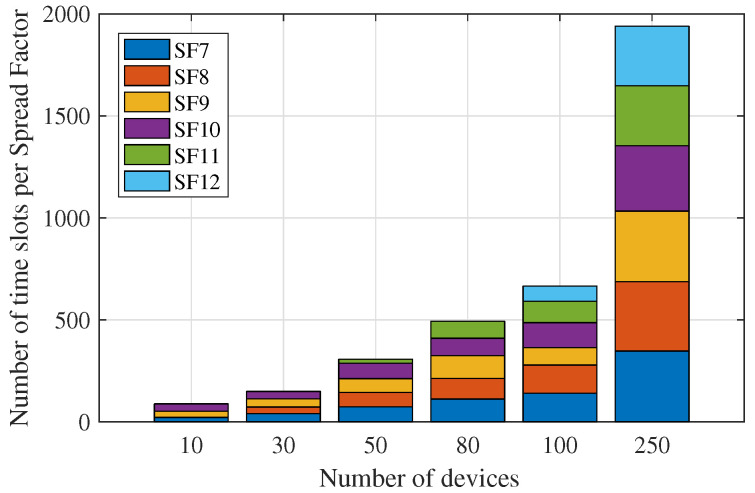
Number of time slots per SF in the considered scheduling.

**Figure 11:**
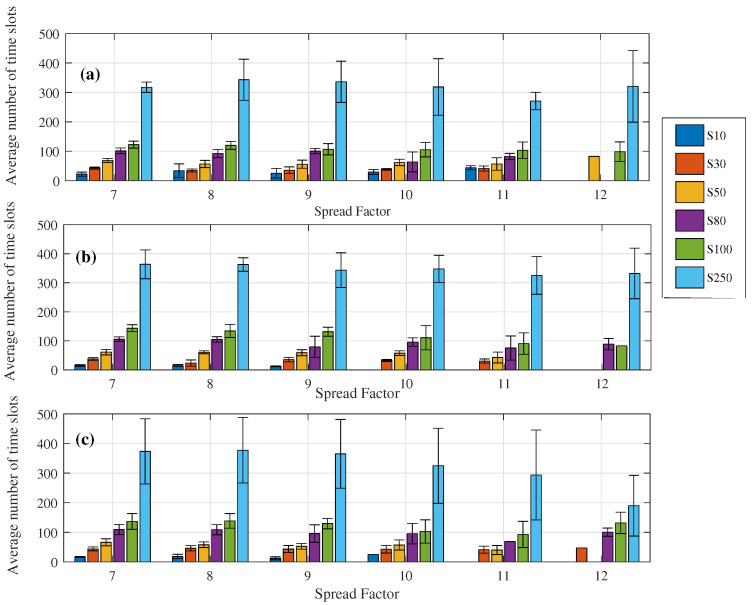
Number of average time slots per SF in the schedule referring to communication between the primary repeater and the gateway, separated by the number of nodes in the network. (**a**) Network #1, (**b**) Network #2, (**c**) Network #3.

Here we assumed that time was divided into slots of 10 ms. Moreover, the implemented scheduling programming considered a matrix with six lines, in which each line represents an SF and each column a time slot. Therefore, the role of the optimization was to scale the devices, aiming at the best allocation with a minimized total data collection time. To evaluate the performance of the algorithm, we only consider the uplink communication between the primary repeater and the gateway. This choice was made because, from the gateway point of view, it can be seen as one of two different levels. Beyond a conventional level 2 device, it can be seen as a level 3 device when there is no end-device connected to it. Thus, this evaluation sought to cover the different ways the modeled network devices can be seen by their respective communication pairs. The evaluation of the scheduling of the modeled network devices was performed by observing the number of time slots assigned by the algorithm to each SF, in a given schedule, as shown in Figure 10 and Figure 11.

It can be observed from Figure 10 and Figure 11 that the algorithm systematically allocates devices in each SF with minimized total data collection time, through equal distribution of the number of time slots per SF. In addition, there is a preference for the use of smaller SFs aiming at a reduction in energy consumption, making use of larger SFs only when network extensions are required. For the networks with the number of nodes equal to 100 and 250, the scaling of the devices became an evident problem and the use of larger SFs is also demanded to preserve the total collection time.

## 7. Results and Discussion

After the evaluation of the proposed optimization methodology in several scenarios, we can summarily emphasize that the greater the number of devices in the network, the greater the total average energy consumed by the devices, and the use of larger values of spread factor is demanded. The results also show that, in general, the required transmission power increases with the decrease in the number of devices because the link distances are relatively large in networks with few elements. Moreover, we could observe that the algorithm systematically allocates devices with minimized total data collection time, through equal distribution of the number of time slots per spread factor. There is a preference for smaller spread factors to minimize energy consumption, and larger values of this parameter are required when network extensions are mandatory.

## 8. Conclusions and Future Works

Performance evaluations of a resource optimization methodology in LoRaWANs were conducted in this work, after model adjustments demanded by practical results previously obtained in coverage experiments. Using variable neighborhood search and a minimum-cost spanning tree algorithm, the proposed procedure allows a reduction in the implementation and maintenance costs of LoRaWANs that employ data repeaters to increase coverage. The joint strategy allowed the minimization of the total energy per useful bit of the network, as well as of the total data collection time, and scaling parameters such as spread factor, bandwidth, and transmission power.

The numerical results show that to reduce the total energy per useful bit, there is a preference for small values of spread factor in networks with few associated devices, whereas, in extended coverage LoRaWANs, the priority is for large values. The results also show that the device scaling over time is an important parameter in the optimization due to the required equality in the distribution of time slots per spread factor and, due to the avoidance of discrepancies in the total time data collection. These achievements pave the way towards LoRaWAN extensions with the employment of LoRa repeaters if the resource allocation optimization proposed in this paper is effectively applied to minimize implementation and maintenance costs. The inclusion of the channel coding parameter in the optimizations can be advantageous since its impact on energy per bit and bit rate can contribute to the device scaling. An investigation of the impact of networks generated with different Graph variants is also left for future work, as well as experimental demonstrations in an apparatus equipped with software that allows the configuration of optimized parameters.

## Figures and Tables

**Table 1:** Parameters configured in the LoRa devices.

Parameter	Symbol	Value	Unit
Transmitted Power	$P_{t}$	−4 to 20	dBm
Carrier Frequency	$f_{c}$	137; 198; *…*; 1020	MHz
Bandwidth	$B_{w}$	7.8 to 500	kHz
Spreading Factor	SF	7; 8; 9; 10; 11; 12	-
Code Rate	CR	4/5; 4/6; 4/7 or 4/8	-

## Data Availability

Not applicable.
